# The longitudinal association between psychotic experiences, depression and suicidal behaviour in a population sample of adolescents

**DOI:** 10.1007/s00127-015-1086-2

**Published:** 2015-07-11

**Authors:** Sarah A. Sullivan, Glyn Lewis, David Gunnell, Mary Cannon, Becky Mars, Stan Zammit

**Affiliations:** Centre for Academic Mental Health, School of Social and Community Medicine, University of Bristol, Bristol, UK; Mental Health Sciences Unit, University College London, London, UK; Department of Psychiatry, Royal College of Surgeons in Ireland, Dublin, Ireland; Institute of Psychological Medicine and Clinical Neurosciences, University of Cardiff, Cardiff, UK; CLAHRC West, School of Social and Community Medicine, University of Bristol, Bristol, UK

**Keywords:** Psychotic experiences, Depressive symptoms, Suicidal behaviour, Epidemiology

## Abstract

**Purpose:**

Whilst psychotic experiences are associated with suicidal behaviour in a number of studies the value of psychotic experiences for the prediction of suicidal behaviour and the role of depressive symptoms in this relationship is not clear. We examined the association between psychotic experiences and subsequent suicidal behaviour and examine the role of depressive symptoms in this relationship.

**Methods:**

Psychotic experiences and depressive symptoms at age 12 and 16 years, and suicidal behaviour at age 16 years were assessed in participants (prospective analysis *n* = 3171; cross-sectional analysis *n* = 3952) from a population-based cohort.

**Results:**

Psychotic experiences (OR 1.75 95 % CI 1.20, 2.54) and depression (OR 3.97 95 % CI 2.56, 6.15) at 12 years were independently associated with suicidal behaviour at 16 years after adjustment for confounding. There was no evidence that the relationship between psychotic experiences and suicidal behaviour was stronger in participants who were also depressive. A ROC analysis showed that adding information on psychotic experiences to measures of depressive symptoms had hardly any effect on improving prediction of suicidal behaviour (AUC increased from 0.64 to 0.65). Whereas adding a measure of depressive symptoms to the measure of psychotic experiences improved prediction substantially (AUC 0.56–0.65).

**Conclusions:**

Psychotic experiences and depression are independently associated with suicidal behaviour although the association with depression is substantially stronger. Psychotic experiences alone are not a strong predictor of later suicidal behaviour and add little to predicting the risk of suicidal behaviour over and above the information provided by depressive symptoms.

## Introduction

Suicide is a major public health issue and results in approximately 800,000 deaths worldwide annually [1]. Schizophrenia is associated with a greatly increased risk of suicide [2]. Whilst the lifetime risk of schizophrenia is approximately 0.7 %, psychotic experiences such as hallucinations and delusions are much more common in the general population [3], though the role of psychotic experiences on suicide risk in general population (i.e. non-clinically ascertained) samples has, until recently, received little attention.

A number of studies using cross-sectional data have demonstrated an association between psychotic experiences and suicidal behaviour in both general population [4–8] and clinical [9, 10] samples. Whilst most of these studies reported associations of moderate effect size, one study [8] reported a very strong association (OR 10.23 95 % CI 3.25, 32.26) between psychotic experiences and suicidal behaviour, suggesting that further study of the role of psychotic experiences on influencing suicide risk should be a priority for suicide prevention research. Psychotic experiences were also associated with a 20-fold increase in risk of suicidal plans or acts in adolescents who also had suicidal thoughts however, these estimates were based on small samples and were therefore imprecise. The majority of adolescents with suicidal plans or acts in that study endorsed psychotic experiences, indicating that suicidal behaviour might also serve as a useful marker for the presence of psychotic experiences, as well as psychotic experiences indexing a high risk of suicidal behaviour.

To the best of our knowledge only one previous longitudinal study [11] has investigated the association between psychotic experiences at baseline and subsequent suicide attempts. Data on psychotic symptoms were collected using semi-structured interviews in a sample of 1112 Irish adolescents and examined in relation to attempted suicide 3 and 12 months later. Of the adolescents who had attempted suicide at the 12-month follow-up 20 % had reported psychotic symptoms at baseline compared with 2.5 % who had not attempted suicide at follow-up. Strong associations were reported at both follow-up time-points (ORs approximately 10). The association between psychotic symptoms and suicide attempts was stronger in those who also had other psychopathologies (ORs approximately 18 and 33), though a lack of precision due to the small number of adolescents with psychotic symptoms who attempted suicide (OR 32.67 95 % CI 10.42, 102.41) make these findings difficult to interpret.

Whilst psychotic experiences might be a strong marker for future suicidal behaviour, depressive symptoms frequently co-occur with psychotic experiences. It is not known whether assessing for psychotic experiences is as useful a predictor of suicide risk as assessment of other psychopathologies, such as depression, that are more commonly assessed during childhood and adolescence. Understanding the role of psychotic experiences in the aetiology of suicidal behaviour, and their predictive utility independent of depression may have important implications for suicide prevention.

In this study we examine whether psychotic experiences in early adolescence are associated with an increased risk of incident suicidal behaviour during mid-adolescence, and whether this is explained by co-morbid depressive symptoms or other confounders. We also examine the potential utility of measures of psychotic experiences as a predictor of suicidal behaviour, and compare this with measures of depressive symptoms and gender that also index risk for suicidal behaviour.

## Materials and methods

### The Avon longitudinal study of parents and children (ALSPAC)

14,541 Pregnant women resident in Avon, UK with expected dates of delivery 1st April 1991 to 31st December 1992 were recruited. Of these initial pregnancies, there was a total of 14,676 foetuses, resulting in 14,062 live births and 13,988 children who were alive at 1 year of age. When the oldest children were approximately 7 years of age an additional 713 children were enrolled resulting in a total sample size of 15,458 foetuses. Of this total sample 14,701 were alive at 1 year.

The cohort is representative of those born in the former county of Avon during this period [12].

Parents of the study children have been completing regular postal questionnaires about their child’s health and development since birth. In addition, all the children have been invited to attend annual assessment clinics since the age of 7 years. At these clinics, various psychological and physical assessments were conducted through face-to-face interviews.

Please note that the study website contains details of all the data that is available through a fully searchable data dictionary http://www.bris.ac.uk/alspac/researchers/data-access/data-dictionary//.

### Dataset

From the original sample of 147,016,606 provided data on psychotic experiences at age 12 years and 4731 at 16 years. Three thousand one hundred and seventy-one participants who had provided data on psychotic experiences and depressive symptoms at age 12 years and suicidal behaviour at age 16 years were included in the prospective analysis and 3952 who had provided psychotic experiences, depressive symptoms and suicidal behaviour data at age 16 were included in the cross-sectional analysis.

### Ethics

Ethical approval for this study was obtained from the ALSPAC Law and Ethics Committee and the Local Research Ethics Committees.

## Measures

### Outcomes at age 16 years

Suicidal behaviour was assessed with a self-report postal questionnaire [13]. The questions were based on those from a European study on child and adolescent self-harm [14].

Our primary definition of suicide attempts was derived by an endorsement to the question “Have you ever hurt yourself on purpose in any way” and the reason given for this by the respondent was that he or she wanted to die (referring to the most recent occasion) or they had ever seriously wanted to kill themselves on any self-harm occasion.

Our secondary definition (suicidal ideation) also investigated suicidal thoughts and plans; it included endorsement of the question above on suicidal self-harm or a question on suicidal plans (1) “Have you ever made plans to kill yourself” or a question on suicidal thoughts (2) “Have you ever thought of killing yourself even if you would not really do it”.

### Psychotic experiences at age 12 years

Psychotic experiences were recorded using the Psychosis-Like Symptom interview (PLIKSi) a semi-structured interview [15] that draws on the principles of standardised clinical examination developed for the Schedule for Clinical Assessment in Psychiatry (SCAN) and asks about experiences over the previous 6 months. There are 11 ‘core’ questions eliciting key psychotic experiences, covering hallucinations (visual and auditory), delusions (spied on, persecution, thoughts being read, reference, control, and grandiosity), and experiences of thought interference (broadcasting, insertion and withdrawal). Any unspecified delusions elicited were also rated. Cross-questioning was used to establish the presence of symptoms, and coding of items following the glossary definitions and rating rules for SCAN. Interviewers were psychology graduates who received training in assessment of the SCAN Psychosis Section and in using the PLIKSi. Interviewers rated symptoms as not present, suspected, or definitely present. Unclear responses after probing were always ‘rated down’, and symptoms only rated as definite when a credible example was provided. Interviewers discussed cases with a psychiatrist if it was unclear how an experience should be rated. At regular intervals interviewer ratings for a sample of recorded interviews were also rated by a psychiatrist to ensure interviewers were rating experiences correctly. In this analysis a binary variable (no vs suspected/definite experiences) was used. In this analysis only experiences which were not attributed to the use of alcohol or recreational drugs, when falling asleep/waking up or due to a fever were rated as being suspected or definitely present.

The average inter-rater reliability across all interviewers was ‘very good’ (Kappa = 0.72) and the average Kappa for test–retest reliability was 0.48, which indicates ‘fair agreement’.

In order to allow the investigation of the association between individual symptoms and suicidal behaviour we created a four category variable; no psychotic experiences, hallucinations only, delusions only, both hallucinations and delusions.

### Psychotic-like experiences at 16 years

Psychotic-like experiences were collected using a self-report postal questionnaire which was based on the psychotic experiences interview described above. The questionnaire asks about the presence, level of conviction (definitely or maybe) and frequency over the past year (none, less than 1/month, more than 1/month). Visual and auditory hallucinations and four types of delusions (being spied on, others reading their thoughts, being sent special messages and being controlled by special powers) were enquired about. For the cross-sectional analysis in this study a binary variable was used (i.e. no psychotic-like experiences vs hallucinations reported as definitely present or a delusional belief reported as definitely present and occurring at least 1/month) [16]. A stricter defining criteria for delusional beliefs was used in order to increase the validity of questionnaire-assessed delusions [17]. For the purposes of this analysis only experiences which could not be attributed to the recent use of alcohol or recreational drugs, when falling asleep/waking up or due to fever were included in the analysis.

In order to investigate the separate associations between hallucinations and delusions and suicidal behaviour as a supplementary analysis, a four category variable was defined as described above.

### Depressive symptoms at 12 and 16 years

Self-reported depressive symptoms were assessed using the Short Moods and Feeling Questionnaire (SMFQ) [18] which asked about symptoms over the previous 2 weeks. The standard version consists of 13 questions but the version used in this analysis consists of only 12. Question 4 “teenager felt restless” was not used because previous work has found that this question was poorly understood [19]. The possible score range for each participant was 0 to 24. To enable comparison with the psychotic experiences variable a dichotomised variable was also created with a cut-off at a score of over 11 to create depressed or not depressed categories. This cut-off has been previously used [20].

### Confounding variables

All analyses were adjusted for gender and social class. Social class was defined as the highest of the mother’s or father’s social class during pregnancy and was categorised using current or last occupation. We also adjusted for self-reported depressive symptoms at age 12 years in the analysis with psychotic experiences as the exposure and for psychotic experiences at age 12 years in the analysis with depressive symptoms as the exposure.

### Statistical analysis

Logistic regression models were used to investigate cross-sectional (age 16) and longitudinal (ages 12–16) associations between psychotic experiences and depression as exposures, and suicidal behaviour as the outcome. Multi-variable models were used to adjust for confounders.

We also investigated whether the association between psychotic experiences and suicidal behaviour differed in those with and without depression by conducting Mantel–Haenszel tests and investigating homogeneity of the ORs in each strata.

Positive likelihood ratios were calculated to give an indication of the clinical utility of early adolescent psychotic experiences and depressive symptoms for predicting suicidal behaviour in mid-adolescence.

In order to investigate the effect of suicidal thoughts on the association between psychotic experiences and depression on suicide attempts we also repeated the analysis above in those who did and did not also have suicidal thoughts (i.e. stratified by the response to the question which asks about suicidal thoughts—see “Methods”).

Receiver operating characteristics (ROC) analyses were used to investigate and compare the utility of depressive symptoms and psychotic experiences at age 12 for predicting suicidal behaviour at 16 years. For this analysis we used a continuous score of depressive symptoms, and a cumulative score of the number of psychotic experiences, weighted according to whether experiences were rated by the interviewer as definitely present or suspected (weightings of 1 and 0.5, respectively; score range 0–12).

As supplementary analyses we investigated the associations between suicidal behaviour and hallucinations, delusions and hallucinations and delusions occurring together using the variable described above. We also investigated a dose response effect by investigating the association between suicidal behaviour and the number of psychotic experiences.

## Results

### Descriptive data

Compared to ALSPAC participants omitted from our analyses due to missing data, participants included in this study were more likely to be female (58.8 vs 46.4 %) and less likely to have suspected or definite psychotic experiences at age 12 years (12.0 vs 14.6 %) and at 16 years (12.1 vs 15.7 %); however there was little difference in depressive symptom scores at age 12 years (mean 4.0 vs 3.8;) and a small difference at 16 years (mean 6.4 vs 5.8). Those not included in the dataset were also more likely to report suicidal behaviour at age 16 years (suicide attempts 8.5 vs 6.3 %; suicidal ideation 18.6 vs 16.5 %).

In our dataset 199 (6.3 %) met criteria for suicide attempts and 523 (16.5 %) met criteria for suicidal ideation.

Table 1 shows that of those with suicide attempts at age 16 23.1 % had psychotic experiences and 16.6 % met criteria for depression at age 12 compared to 11.2 and 3.8 %, respectively in those without suicidal behaviour.

**Table 1:** Frequencies of exposures at age 12 (*n* = 3171) and age 16 (*n* = 3952) and covariates by suicidal behaviour categories

Exposure	Suicidal behaviour			
	Suicide attempts		Suicidal ideation	
	No *n* (%)	Yes *n* (%)	No *n* (%)	Yes* n* (%)
Psychotic experiences at 12 years				
None	2638 (88.8)	153 (76.9)	2366 (89.4)	425 (81.3)
Suspected/definite	334 (11.2)	46 (23.1)	282 (10.7)	98 (18.7)
Psychotic experiences at 16 years				
None	3452 (93.5)	190 (73.1)	3099 (94.3)	543 (81.5)
Suspected/definite	240 (6.5)	70 (26.9)	187 (5.7)	123 (18.5)
Depressive symptoms at 12 years				
Not depressed	2860 (96.2)	166 (83.4)	2572 (97.1)	454 (86.8)
Depressed	112 (3.8)	33 (16.6)	76 (2.9)	69 (13.2)
Depressive symptoms at 16 years				
Not depressed	3240 (87.8)	107 (41.2)	3006 (91.5)	341 (51.2)
Depressed	452 (12.2)	153 (58.9)	280 (8.5)	325 (48.8)
Gender				
Male	1267 (42.6)	39 (19.6)	1175 (44.4)	131 (25.1)
Female	1705 (57.4)	160 (80.4)	1473 (55.6)	392 (75.0)
Social class				
High	1020 (34.3)	58 (29.1)	1009 (34.3)	69 (29.9)
Low	1952 (65.7)	141 (70.9)	1931 (65.7)	162 (70.1)

*Suicide attempts* suicidal self-harm, *suicidal ideation* suicidal self-harm, suicidal thoughts or suicidal plans

### Associations between psychotic experiences, depressive symptoms and suicidal behaviour

#### Psychotic experiences at 12 years and suicide attempts at 16 years

In unadjusted models psychotic experiences at 12 years were associated with an approximate 2.4-fold increase in the odds of suicidal behaviour at 16 years (Table 2). There was no evidence that this association differed in those with and without depression at 12 years (*p* = 0.48). After adjusting for depressive symptoms at 12 years and gender and social class the association was attenuated to an approximate 1.7-fold increase.

**Table 2:** Associations (ORs and 95 % CIs) between psychotic experiences and depression at 12 (*n* = 3171) and 16 years (*n* = 3952) and suicidal behaviour at 16 years

	Unadjusted			Adjusted^b^			Homogeneity *p* ^c^	Homogeneity *p* ^f^
	OR	95 % CI	*p* value	OR	95 % CI	*p* value		
Suicide attempts^a^								
Psychotic experiences (age 12)^d^	2.37	1.68, 3.37	≤0.0001	1.75	1.20, 2.54	≤0.01	0.48	0.29
Depression (age 12)^e^	5.08	3.34, 7.71	≤0.0001	3.97	2.56, 6.15	≤0.01		
Psychotic experiences (age 16)^d^	5.30	3.91, 7.18	≤0.0001	2.58	1.81, 3.69	≤0.01	0.05	
Depression (age 16)^e^	10.25	7.86, 13.37	≤0.0001	7.86	5.97, 10.37	≤0.01		
Suicidal ideation^a^								
Psychotic experiences (age 12)^d^	1.93	1.50, 2.49	≤0.0001	1.31	0.99, 1.73	0.06	0.54	
Depression (age 12)^e^	5.14	3.66, 7.23	≤0.0001	4.36	3.07, 6.20	≤0.01		
Female gender	2.38	1.91, 2.97	≤0.0001	2.31	1.85, 2.89	≤0.01		
Psychotic experiences (age 16)^d^	3.75	2.93, 4.70	≤0.0001	2.00	1.48, 2.71	≤0.01	0.36	
Depression (age 16)^e^	10.23	8.41, 12.44	≤0.0001	8.73	7.15, 10.67	≤0.01		

OR odds ratio, *CI* confidence interval

^a^Suicidal ideation includes suicidal thoughts and plans, suicide attempts do not include suicidal thoughts or plans

^b^Adjusted gender + depression at 12/psychotic experiences at 12 + social class (highest of mother’s and father’s)

^c^Mantel–Haenszel test of homogeneity across strata of depression

^d^Suspected or definite

^e^Score cut-off >11

^f^Mantel Haenszel test of homogeneity across strata of suicidal ideation

The positive likelihood ratio (PLR) indicated that psychotic experiences at 12 were a moderately strong predictor of suicidal behaviour at 16 years (PLR 1.93 95 % CI 1.52, 2.45) and the AUC for the psychotic experiences score predicting suicidal behaviour from the receiver operating characteristics (ROC) analysis was 0.56.

There was no evidence that the association between suicide attempts and psychotic experiences differed in those with and without suicidal thoughts (*p* = 0.29).

The fully adjusted results of the supplementary analysis did not indicate any true difference in the strength of the associations between suicidal attempts and hallucinations, delusions or both hallucinations and delusions (results available on request). Our results suggested evidence of a dose–response relationship between the number of psychotic experiences and suicidal attempts (results available on request).

#### Depression at 12 years and suicide attempts at 16 years

In unadjusted models depression at age 12 was associated with an approximately five-fold increase in the odds of suicidal behaviour at 16 years. After adjusting for psychotic experiences at 12 years and other confounders the association was attenuated to a four-fold increase in odds.

The PLR indicated that depression at 12 was a strong predictor of suicidal behaviour at 16 years (PLR 4.25 95 % CI 3.11, 5.81) and the ROC analysis indicated that depressive symptoms increased the probability of correctly predicting suicidal behaviour from 0.5 (chance) to 0.64.

**Fig. 1:**
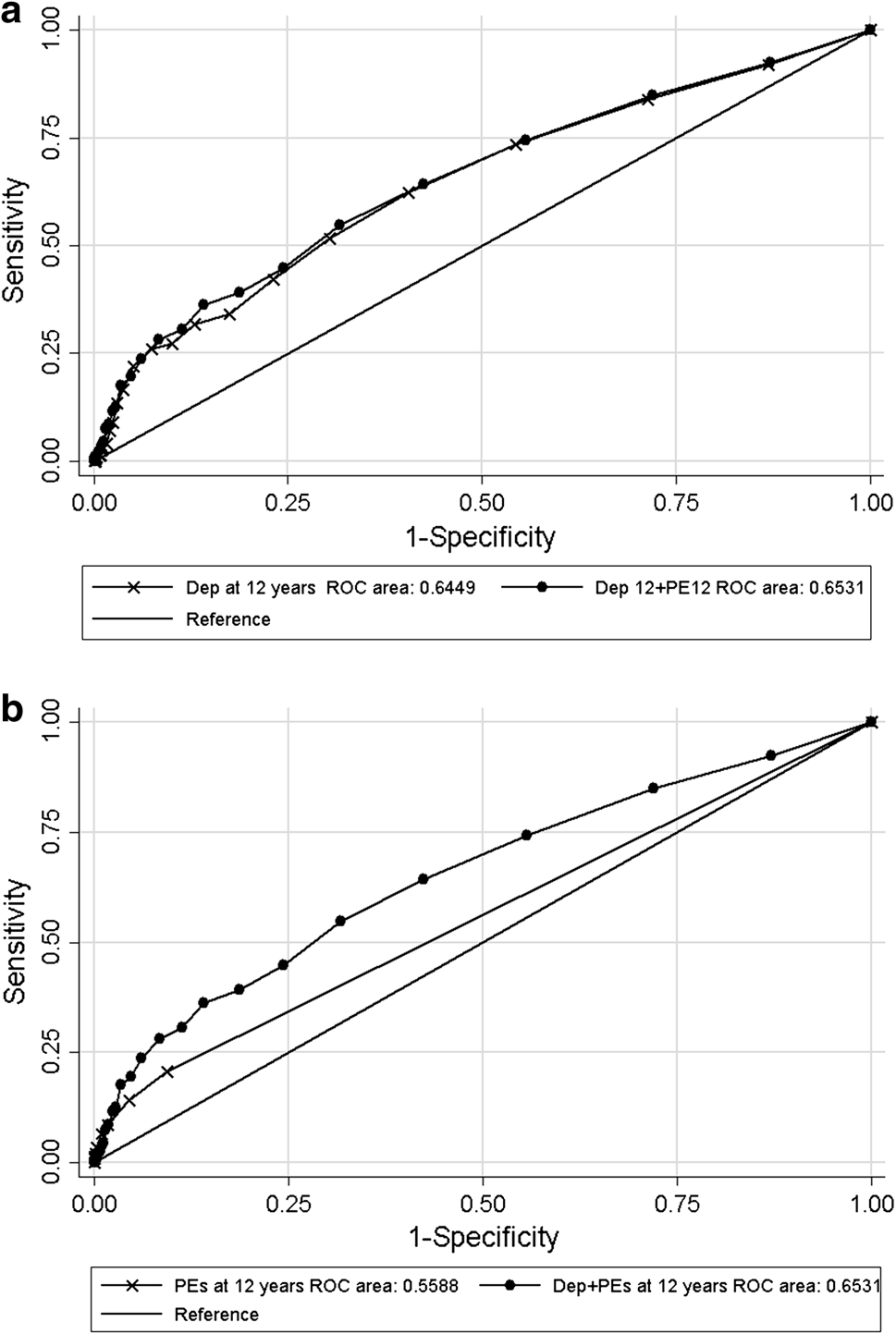
**a** ROC analysis of suicide attempts and psychotic experiences at 12 years and depressive symptoms + number of psychotic experiences at 12 years. **b** ROC analysis of suicide attempts and depressive symptoms at 12 and depressive symptoms + number of psychotic experiences at 12 years

Adding the measure of psychotic experiences to the depressive symptoms score made little difference to the predictive value for suicidal behaviour (AUC increased from 0.64 to 0.65 *p* = 0.02 testing the null hypothesis that the predictive value of depressive symptoms alone is the same as depressive symptoms plus psychotic experiences) (see Fig. 1a). Adding the depression score to the measure of psychotic experiences made a much more substantial difference to the predictive value for suicidal behaviour (AUC increased from 0.56 to 0.65 *p* ≤ 0.001 testing the null hypothesis that the predictive value of psychotic experiences alone is the same as psychotic experiences plus depression score) (see Fig. 1a, b).

Furthermore, the AUC for gender predicting suicidal behaviour was 0.62. Psychotic experiences at age 12 were therefore less informative for predicting suicidal behaviour than gender.

### Cross-sectional associations

Table 1 shows that of those with suicide attempts at age 16 26.9 % had psychotic experiences and 58.9 % met criteria for depression at age 16 compared to 6.5 and 12.2 %, respectively in those without suicidal behaviour.

#### Psychotic experiences and suicide attempts at 16 years

In unadjusted associations psychotic experiences were associated with an approximate five-fold risk of suicidal behaviour, this association attenuated after adjustment to an approximate 2.6-fold risk. There was marginal evidence that this association differed in those with and without depression at 16 years (*p* = 0.05) (see Table 2).

The fully adjusted supplementary analysis showed no evidence of a difference in the strength of the associations between hallucinations, delusions and both hallucinations and delusions at age 16 and suicide attempts at the same age (results available on request).

#### Depression and suicide attempts at 16 years

Before confounder adjustment depression was associated with an approximate ten-fold increase in risk of suicidal behaviour, this association attenuated to an approximate eight-fold increase in risk after adjustment (see Table 2).

### Sensitivity analysis

#### Suicidal ideation

When the analyses were repeated using suicidal ideation as the outcome there was weaker evidence for an association with psychotic experiences at 12 years (adjusted OR 1.31 95 % CI 0.99, 1.73) whereas the association with depression remained strong (adjusted OR 4.36 95 % CI 3.07, 6.20). There was no evidence that the association between psychotic experiences and suicidal behaviour differed across strata of people with and without depression at 12 years (*p* = 0.54). The AUC for depressive symptoms correctly predicting suicidal behaviour was 0.67 and for psychotic experiences was 0.54.

## Discussion

### Main findings

Early adolescent psychotic experiences and depression were both associated with mid adolescent suicidal behaviour. However, once mutually adjusted for the other psychopathology and other confounders, the association between psychotic experiences and suicidal behaviour was attenuated by approximately 36 %, whereas the association between depression and suicidal behaviour was attenuated by approximately 15 %. A similar pattern was observed for cross-sectional associations between depression and psychotic experiences and suicidal behaviour in mid-adolescence, although evidence for an association between psychotic experiences and suicidal behaviour was stronger than in the longitudinal analysis. The cross-sectional association between psychotic experiences and suicidal behaviour that we observed was weaker than that found in previous cross-sectional studies with clinical participants [9, 10], though similar to that reported in two other general population studies [4, 6]. This is not unexpected given that psychotic experiences in individuals from a general population sample and psychotic disorder in individuals from a clinical sample are likely to lie at different locations on the psychosis continuum. Alternatively, the association detected in studies with clinical participants could be an example of Berkson’s bias. Attendance at a clinic may be related both to psychotic experiences and suicidal behaviour. A non-causal association may therefore have been erroneously introduced [21]. Our effect size was substantially smaller than those reported in one cross-sectional population-based study, and in a previous longitudinal study, both of which reported approximately 10-fold increases in odds of suicidal behaviour in those with psychotic experiences compared to those without [8, 11]. Even at the upper-most limit of our confidence intervals, our results were not compatible with a more than four-fold increase in odds of suicidal behaviour in those with psychotic experiences. In comparison, the effect sizes we observed between depression and suicidal behaviour were large, with an approximately eight-fold increase in suicidal behaviour in those with depression compared to those without depression at age 16, and a four-fold increase in the longitudinal analysis.

There are several possible reasons for the large difference in the strength of the association detected between psychotic experiences and suicidal behaviour in our study and in the studies above, including the use of different instruments for measuring psychotic experiences and suicidal behaviour, different methods of data collection (i.e. self-report and semi-structured interviews), different informants (parent and adolescent), confounding and a shorter follow-up period in the case of the longitudinal study [11]. For instance one of the studies [8] used extremely detailed interviews to measure psychotic experiences, suicidal behaviour and depressive symptoms, whereas in our study self-report measures were used to collect information on all of these variables at 16 years. Additionally depression was measured as both lifetime and over the previous month, whereas in our study questions about depressive symptoms just covered the previous 2 weeks. It is possible that these more detailed measures increased the sensitivity in this study [8] and therefore stronger associations were found. Also in the longitudinal study Kelleher study [11] suicidal behaviour was assessed using much more detailed questions than in our study which also may have increased sensitivity and therefore effect sizes. A further possibility is that the effect size is over-estimated in the two studies which reported the strongest associations; it is recognised that studies with small samples and low statistical power are more likely to suffer from effect inflation [22].

Furthermore, whilst the effect of psychotic experiences on suicidal behaviour was described as being strongest within a subgroup of those with depression and with suicidal thoughts in one study [8], we found no evidence in the prospective analysis that the association between psychotic experiences and suicidal behaviour differed in those who were also depressed compared with those who were not or in those who had suicidal thoughts compared to those who did not.

The PLRs and the ROC tests also indicated that depressive symptoms were a substantially better predictor of future suicidal behaviour than psychotic experiences. Furthermore, whilst depressive symptoms at 12 had a probability of correctly predicting 64 % of future suicidal behaviour, adding information on psychotic experiences hardly altered this, whereas whilst psychotic experiences at 12 had a probability of correctly predicting 56 % of future suicidal behaviour, adding depressive symptoms substantially increased the probability of prediction.

### Implications

If we had replicated the very strong associations found in previous studies [23, 24] this would have suggested that psychotic experiences in early adolescence are a very strong indicator of suicidal behaviour both at the time and in later adolescence. However our results call into question the utility of psychotic experiences as a predictor of later suicidal behaviour. Our findings suggest that screening for psychotic experiences at 12 years of age tell us little about suicide risk at age 16; information on gender was more predictive of suicidal behaviour, whilst a measure of depressive symptoms at age 12 was the most useful for prediction of suicidal risk. Whilst clinicians should investigate whether those with psychotic experiences also have suicidal thoughts, assessing depressive symptoms is of more importance when assessing suicidal risk. The area under the curve (ROC) analysis shows that adding information about psychotic experiences to that of depressive symptoms infers little added benefit when addressing future suicidal behaviour risk whereas adding information on depressive symptoms is highly informative when added to information on psychotic experiences.

The association observed between psychotic experiences and suicidal behaviour in other studies is likely to be, at least partly, due to co-morbid depressive symptoms since there is convincing evidence of the strong overlap between psychotic experiences and depressive symptoms [25, 26] and our results showed the association between psychotic experiences and suicidal behaviour attenuated substantially after adjusting for depressive symptoms. Suicidal behaviour may also result from particular psychotic experiences such as command hallucinations or delusional thought processes that drive forward this behaviour, although this explanation seems less likely in non-clinical populations.

Whilst psychotic experiences in early adolescence seem to have limited value for predicting suicidal behaviour in mid-adolescence suicidal behaviour does index likelihood of having psychotic experiences to some extent. In our dataset 27 % of those who had attempted suicide also had psychotic-like experiences at that time compared to 6 % of those without suicidal behaviour.

### Strengths and limitations

The prospective design of our study is a strength which has enabled us to determine the utility of psychotic experiences at predicting suicidal behaviour over time in a large general population sample of adolescents that is broadly representative of the UK adolescent population. Furthermore this is one of the largest samples available to address this question with longitudinal data in such a well characterised cohort.

Selection bias due to participant attrition is a limitation of our study since data were missing for a substantial proportion of the cohort. Those not included in the analysis were more likely to have had psychotic experiences and suicidal behaviour indicating that the effect sizes that we detected might have been larger in the absence of participant attrition.

A previous study has shown that attrition within ALSPAC tends to have little effect on estimates of association [27] and it seems unlikely that attrition has led to substantial distortion of the associations between psychotic experiences, depression and suicidal behaviour in this cohort.

Since the questions about suicidal behaviour asked about lifetime experience it is possible that in the cross-sectional analysis suicidal behaviour and psychotic experiences may not have been concurrent, although others [8] have found that positive responses to questions about psychotic experiences in adolescence nearly always refer to very recent experiences.

Only psychotic experiences which were not attributed to the effects of alcohol or drug use, falling asleep/waking up or the effects of fever are used in this analysis and therefore the findings are not likely to be confounded by these factors.

## Conclusion

The positive likelihood ratio indicates that the predictive power for early adolescent depressive symptoms was more than twice as strong as that for early adolescent psychotic experiences, whilst the ROC analysis shows that information on psychotic experiences adds very little to depressive symptoms for predicting later suicidal behaviour. This suggests that although clinicians should be aware that early adolescent psychotic experiences may infer an added risk of later suicidal behaviour, screening for psychotic experiences as well as for depressive symptoms is likely to be of limited value for identifying adolescents at greater risk of suicidal behaviour in the general population.
